# Iron Oxide Nanoparticles as an Alternative to Antibiotics Additive on Extended Boar Semen

**DOI:** 10.3390/nano10081568

**Published:** 2020-08-10

**Authors:** Ioannis A. Tsakmakidis, Theodoros Samaras, Sofia Anastasiadou, Athina Basioura, Aikaterini Ntemka, Ilias Michos, Konstantinos Simeonidis, Isidoros Karagiannis, Georgios Tsousis, Mavroeidis Angelakeris, Constantin M. Boscos

**Affiliations:** 1 School of Veterinary Medicine, Faculty of Health Sciences, Aristotle University of Thessaloniki, 54627 Thessaloniki, Greece; anastasiadousof@yahoo.gr (S.A.); basioura@vet.auth.gr (A.B.); demkaterina@hotmail.com (A.N.); imichos84@hotmail.com (I.M.); anisivet@yahoo.gr (I.K.); tsousis@vet.auth.gr (G.T.); pboscos@vet.auth.gr (C.M.B.); 2 School of Physics, Faculty of Sciences, Aristotle University of Thessaloniki, 54124 Thessaloniki, Greece; theosama@auth.gr (T.S.); ksime@physics.auth.gr (K.S.); agelaker@auth.gr (M.A.)

**Keywords:** bacterial resistance, boar, microbiological analyses, nanoparticles, semen

## Abstract

This study examined the effect of Fe_3_O_4_ nanoparticles on boar semen. Beltsville thawing solution without antibiotics was used to extend ejaculates from 5 boars (4 ejaculates/boar). Semen samples of control group (C) and group with Fe_3_O_4_ (Fe; 0.192 mg/mL semen) were incubated under routine boar semen storage temperature (17 °C) for 0.5 h and nanoparticles were removed by a magnetic field. Before and after treatment, aliquots of all groups were cultured using standard microbiological methods. The samples after treatment were stored (17 °C) for 48 h and sperm parameters (computer-assisted sperm analyzer (CASA) variables; morphology; viability; hypo-osmotic swelling test (HOST); DNA integrity) were evaluated at storage times 0, 24, 48 h. Semen data were analyzed by a repeated measures mixed model and microbial data with Student’s t-test for paired samples. Regarding CASA parameters, Fe group did not differ from C at any time point. In group C, total motility after 24 h and progressive motility after 48 h of storage decreased significantly compared to 0 h. In group Fe, linearity (LIN) after 48 h and head abnormalities after 24 h of storage increased significantly compared to 0 h. The microbiological results revealed a significant reduction of the bacterial load in group Fe compared to control at both 24 and 48 h. In conclusion, the use of Fe_3_O_4_ nanoparticles during semen processing provided a slight anti-microbiological effect with no adverse effects on sperm characteristics.

## 1. Introduction

The performance of artificial insemination (AI) is the most acknowledged method worldwide to fertilize sows with liquid-extended boar semen. Bacterial contamination affects semen’s qualitative characteristics and fertilizing capacity and induces a potential health risk to the females after AI. Previous studies reported that bacteriospermia results in higher estrus returns, early embryo death, endometritis and specific infections in pigs [1,2,3]. Porcine semen usually contains two to three species of bacteria, most commonly *Staphylococci, Streptococci* and *Pseudomonas* [4]. In this aspect, the antibiotics have been main constituents of semen extenders to control the bacterial growth during the storage time of boar liquid semen. However, Althouse and Lu [5] found bacterial occurrence in one third of the produced boar sperm doses, with bacteria largely resistant to antibiotics such as amoxycillin, gentamycin, lincomycin and tylosin. This fact has led the scientific community to explore alternative strategies to minimize the development of antibiotic resistance. Thus, methods of physically removing bacteria by centrifugation in colloidal solutions [6] and the addition of natural or synthetic peptides with antimicrobial activity to the diluents have been reported in boar semen processing [7,8]. Nanoparticles (NPs) of size 40–60 nm, expressed significant antimicrobial capacity against *Escherichia coli*, *Pseudomonas aeruginosa* and *Staphylococcus aureus* [9]. Li et al. [10], investigating the mechanism of action of Ag NPs, found that 10 μg Ag NPs/mL can completely restrain the growth of 10^7^ cfu/mL of *E. coli* cells, by damaging cell membrane structure, limiting the activity of enzymes and inducing bacterial death. The results of the study of Shahverdi et al. [11], where silver NPs significantly increased the antimicrobial activity of vancomycin, amoxycillin and penicillin against *S. aureus* are remarkable. Moreover, iron oxide magnetic NPs provided significant antibacterial properties against *p. aeruginosa* and *S. aureus*, due to the reactive oxygen species (ROS) generation [12,13]. Although the scientific community continuously seeks alternative approaches to the use of antibiotics in semen processing, some studies have reported toxic effects of NPs on live cells. Sahu et al. [14] found that nanotoxicity could be dependent on the type of the cell in terms of a different sensitivity response. For this reason, our research team was the first to investigate the appropriate effective antibacterial concentration and co-incubation time of silver Ag/Fe and Fe_3_O_4_ NPs in semen [15]. In this study, Ag/Fe NPs demonstrated a detrimental effect on boar spermatozoa. Conversely, Fe_3_O_4_ NPs at a minimum inhibitory concentration (0.192 mg/mL semen) had no negative effect on computer-assisted sperm analyzer (CASA) motility parameters of boar sperm after 30 min of co-incubation [15]. Therefore, further research on their application for semen handling is necessary. The aim of this research was to extend our knowledge regarding an alternative methodology in order to control the microbial load of boar semen without having detrimental effects on its quality, using iron oxide NPs.

## 2. Materials and Methods

The semen samples used in the present study were commercially available. No operations on research animals were carried out and no approval by the Ethics Committee on Animal Use of Aristotle University of Thessaloniki (Greece) was necessary.

### 2.1. Reagents and Media

All the reagents and chemicals used were purchased from Sigma Aldrich, Seelze, Germany, unless otherwise stated. Semen samples were extended with laboratory-produced Beltsville thawing solution (BTS: 205 mM glucose, 20.4 mM 112 sodium citrate, 10.0 mM KCl, 15.0 mM NaHCO_3_, 3.6 mM ethylenediaminetetraacetic acid (EDTA); pH 7.2–7.4; 290–300 mOsmol/kg) without antibiotics.

### 2.2. Synthesis and Dispersions of Fe_3_O_4_ NPs

#### 2.2.1. Synthesis

Magnetite nanoparticles were synthesized by the oxidative precipitation of FeSO_4_ in an ethanol/water mixture and NaNO_3_ and NaOH were added as mild oxidant and acidity controller, respectively [16,17]. A solution of 1 M FeSO_4_·7H_2_O was prepared by the reagent’s dissolution in 350 mL of 0.01 M H_2_SO_4_ solution. Another solution with dissolved NaNO_3_ (0.25 M) and NaOH (0.52 M) in a 30% ethanol mixture in distilled water was prepared to a total volume of 1400 mL. The two solutions were mixed under intense stirring to form green rust. After agitating the mixture for 15 min, it was transferred to a water bath regulated at 90 °C and allowed for 6 h to ageing of green rust and to the formation of magnetite nanoparticles. When cooled to room temperature, the dispersion was washed/centrifuged several times with distilled water to remove any residuals. The Fe_3_O_4_ NPs were sterilized before co-incubation with semen by heating in an autoclave (steam sterilization, 121–124 °C, 3 atm, 20 min).

#### 2.2.2. Characterization

The identification of the structural phases appearing in the nanoparticles was carried out by powder X-ray diffractometry (XRD) using an Ultima+ diffractometer, Rigaku, Sendagaya, Japan, operating with CuKα radiation at 40 kV/30 mA, 0.05° for step size and 3 s as step time. The diffraction diagrams were evaluated after comparison with the Powder Diffraction Files (PDF) database [18]. Scanning electron microscopy (SEM) images were taken by a Quanta 200 ESEM FEG instrument (FEI, Hillsboro, OR, USA) with a field-emission gun adjusted to 30 kV. Magnetic measurements of the nanoparticles were performed in a MPMS XL SQUID magnetometer (Quantum Design, San Diego, CA, USA) at room temperature.

To determine the percentage of bivalent iron in the nanoparticles (Fe^2^+/Fe^3^+ ratio) as an indicator of Fe_3_O_4_ formation, the dried sample (0.1 g) was digested in 50 mL 7 M H_2_SO_4_ under heating and then, titrated by a 0.05 M KMnO_4_ solution. The appearance of pink color signified the complete reduction of MnO_4_^−^ ions and the end of the titration. The sum of iron in the sample was defined by graphite furnace atomic absorption spectrophotometry (Perkin Elmer AAnalyst 800, Perkin Elmer, Waltham, MA, USA) after dissolving a weighted quantity in HCl.

### 2.3. Animals, Semen Samples Collection and Dilution

The semen samples were collected from 5 crossbred boars (2–2.5 years of age) from a commercial pig farm with capacity of 700 sows. In total, 20 ejaculates (4 ejaculates/boar) were collected by the gloved hand technique and the gelatinous portion was discarded using a gauze. Two ejaculates were collected on a weekly basis, pooled, and transported in an isothermal vessel (37 °C) to the farm laboratory. The ejaculates were assessed for the basic quality parameters [volume, concentration (SDM1, Minitube^®^, Tiefenbach, Germany) and motility (subjective microscopic evaluation by a phase contrast microscope, Zeiss, Oberkochen, Germany)], and those with volume >200 mL, concentration >200 × 10^6^ sperm/mL, total number of spermatozoa/ejaculate >40 × 10^9^, and gross motility >70% were further processed.

Semen samples of good quality were extended in BTS without antibiotics (30 × 10^6^ spermatozoa/mL) and re-evaluated microscopically for motility. Extended pooled semen samples with motility >70% were transported (17 °C) within 60 min into a portable semen storage unit (Minitube^®^, Tiefenbach, Germany) to the Unit of Biotechnology of Reproduction, Clinic of Farm Animals, Faculty of Veterinary Medicine, Aristotle University of Thessaloniki.

### 2.4. Experimental Design

#### 2.4.1. Semen Processing with Nanoparticles (NPs)

Upon arrival at the Unit of Biotechnology of Reproduction, each semen sample was separated in 2 aliquots and the following two experimental groups were prepared: (1) control group (C): extended semen without any treatment; (2) iron oxide group (Fe): extended semen with Fe_3_O_4_ NPs (0.192 mg Fe_3_O_4_/mL semen).

#### 2.4.2. Trial 1: Determination of Non-Detrimental Co-Incubation Time of Semen with NPs

The beneficial/detrimental co-incubation period and the appropriate antibacterial concentration of iron oxide NPs for boar semen handling, was evaluated in a previous trial of our laboratory [15]. Briefly, semen samples were cultured for the detection of bacterial pathogens. Plates containing sheep blood agar and plate count agar (PCA, Oxoid, Thermo Scientific, Lenexa, KS, USA) were inoculated with 100 μL aliquot of extended semen without antibiotics and incubated at 37 °C to estimate the microbial load and select strains for the antibacterial assay. Isolation and identification of picked colonies from blood agar was performed after incubation in 10 mL of tryptone soya broth (TS broth, Oxoid, Thermo Scientific, Lenexa, KS, USA), (37 °C for 24 h) and the conduction of conventional laboratory procedures. *Pseudomonas*, *Staphylococcus* and *Streptococcus* strains were finally selected for further investigation of the antimicrobial activity of nanoparticles. The selected strains were tested against several concentrations of the examined iron oxide NPs using a standard two-fold broth dilution method [19]. Dilutions of the Fe_3_O_4_ NPs were prepared and dispensed in tubes. Each tube was inoculated with the relevant microbial inoculum (adjusted to 0.5 McFarland scale) of each of the selected strains. Tubes were incubated at 37 °C aerobically and 100 μL were spread on blood agar at time 0, 30 min, 60 min and 24 h of incubation. The results demonstrated that the concentration of iron oxide NPs that prevented the growth of the selected bacteria was different for each strain. The minimum inhibitory concentration (MIC) of NPs for the bacterial growth was defined as the lowest concentration of NPs, which inhibited bacterial growth.

Concentrations of iron oxide NPs were re-assessed and finally selected so as not to be harmful for semen samples. Considering the MIC (0.192 mg/mL semen) of the examined NPs after in vitro antimicrobial activity assessment, they were dissolved in distilled water to prepare a stock solution of 19.2 mg Fe_3_O_4_ NPs/mL distilled water. A fresh stock solution of NPs was prepared every week. Prior to its use, the NPs solution was sonicated for 20 min to improve its dispersion stability. The C and NPs groups were incubated at 17 °C (appropriate storage temperature of extended boar semen) for 30, 45 and 60 min and afterwards the NPs were removed with a magnetic field (as it is described in Trial 2). Total motility and progressive movement spermatozoa were assessed by the CASA. The incubation period of 45 and 60 min were excluded as the values of the examined CASA parameters were significantly decreased in the NPs groups compared to the control group. The co-incubation period of 30 min had no adverse effects on the evaluated CASA parameters and was selected for further research.

#### 2.4.3. Trial 2: Investigation of the Effect of Iron Oxide (Fe_3_O_4_) NPs on Boar Semen Quality

**Figure 1:**
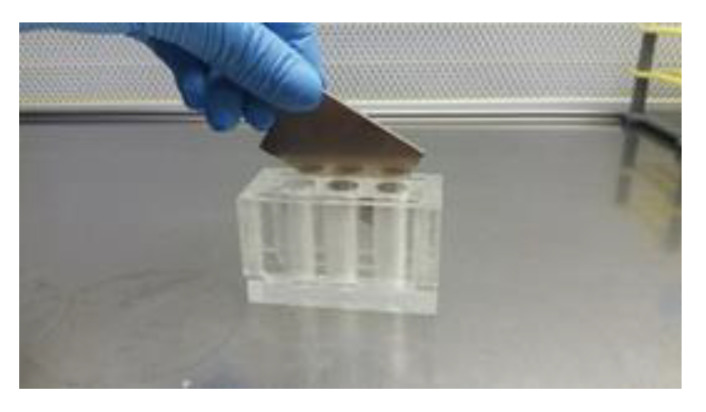
Plexiglass acrylic rack with 6 Eppendorf tubes’ places, equipped with commercial NdFeB permanent magnets.

The C and Fe groups were incubated (17 °C) for 30 min after iron oxide NPs addition to group Fe. Subsequently NPs were removed with the help of a magnetic field. To achieve that, the tubes were placed in a plexiglass acrylic rack equipped with commercial NdFeB permanent magnets, remained in vertical position for at least 5 min and the post-treated semen was transferred to a new tube, while the NPs were discarded (Figure 1). Three repetitions of this process were applied to completely remove the NPs. Then the control and the post-treatment NPs samples were stored at 17 °C for 48 h. Semen quality and functionality as well as the microbiological tests were performed at 0 (time of NPs removal), 24 and 48 h post treatment.

### 2.5. Sperm Kinetics/Motility

The Sperm Class Analyser (SCA^®^, Microptic S.L., Barcelona, Spain) CASA system, a microscope (AXIO Scope A1, Zeiss, Oberkochen, Germany) with a heating stage (37 °C) and a camera (Basler scA780 54fc, Ahrensburg, Germany) were used for the evaluation of sperm motility and kinetics. The analysis by SCA^®^ software (v.6.3.) was performed with the following configurations: 4–6 fields were recorded (×100) for each sample, >500 spermatozoa, 25 frames/s, region of particle control 10–18 microns, progressive movement of >45% of the parameter straightness (STR), circumferential movement <50% linearity (LIN), depth of field 10, and temperature of the microscope plate 37 °C. The debris incorrectly classified as spermatozoa were manually deleted.

A semen sample volume of 10 μL was placed on Makler counting chamber (Makler^®^, 10 μm, Sefi Medical Instruments, Haifa, Israel), which was preheated at 37 °C, and the following parameters were evaluated: (1) total and progressive motility (%), (2) spermatozoa with slow/medium/rapid movement (10 < slow < 25 < medium < 45 < rapid μm/s; %), (3) straight line velocity (VSL; μm/s), (4) curvilinear velocity (VCL; μm/s), (5) average path velocity (VAP; μm/s), (6) linearity (LIN; VSL/VCL × 100), (7) straightness (STR; VSL/VAP × 100), (8) wobble (WOB; VAP/VCL × 100), (9) amplitude of lateral head displacement (ALH; μm), (10) beat cross-frequency (BCF; Hz), and (11) hyperactivation (LIN < 0.32, VSL > 97 μm/s, ALH > 3.5 μm; %).

### 2.6. Sperm Viability

Sperm viability was evaluated applying the eosin-nigrosine staining protocol in one step [20]. Two hundred spermatozoa were estimated in of an optical microscope (×1000; Zeiss, Oberkochen, Germany), to calculate the ratio (%) of live-dead cells.

### 2.7. Sperm Morphology

According to the manufacturer’s instructions, the SpermBlue staining method (SpermBlue^®^, Microptic S.L., Barcelona, Spain) was applied for the assessment of sperm morphology. For each semen sample, 200 spermatozoa were counted microscopically (×400; Zeiss, Oberkochen, Germany) and the results were described as percentage of spermatozoa with normal morphology or with morphological abnormalities including head and integrity of acrosome membrane, midpiece, tail, and cytoplasmic droplets.

### 2.8. Sperm Membrane Functionality

For the sperm membranes functionality, the hypo-osmotic swelling test (HOST) was applied according to Vazquez et al. [21] after modification. The HOST solution was prepared (75 mmol/L fructose; 32 mmol/L sodium citrate) and the osmolarity was adjusted to 150 mosm/kg (OSMOMAT^®^ 030, Gonotec, Berlin, Germany). Briefly, for each group, a semen sample of 100 μL was mixed with 1 mL of HOST solution and incubated (37 °C) for 1 h. Finally, 200 spermatozoa per sample were evaluated microscopically (×400; Zeiss, Oberkochen, Germany) and the results were indicated as spermatozoa with functional membrane that is with swollen tails (%).

### 2.9. Sperm DNA Integrity

Sperm DNA integrity was assessed by the acridine orange test [22], which quantifies the metachromatic shift of acridine orange fluorescence [23]. Specifically, spermatozoa with compact chromatin structure fluoresced green, while those with damaged chromatin integrity fluoresced red. For each semen sample, 200 spermatozoa were counted under a fluorescence microscope (×1000; Zeiss, Oberkochen, Germany) and the results were expressed as spermatozoa with damaged DNA (%).

### 2.10. Microbiological Analysis

Samples from C and Fe experimental groups were subjected to microbiological analysis for bacterial counts and culture using standard protocols. Preparations of all culture media were made according to the manufacturer’s recommendations. Samples were diluted up to 10^−6^ in 0.9% normal saline and 100 μL of each dilution were spread into plate count agar (OXOID) and incubated at 37 °C. The plates were read after 24 and 48 h and the number of colonies formed was reported as colony forming units per mL (cfu/mL). For the detection of frequently isolated bacteria in semen such as *Staphylococcu*s spp., *Streptococcus* spp., *Enterobacter* spp., *Bacillus* spp., *Proteus* spp., *Escherichia coli*, *Pseudomonas aeruginosa* [4], 100 μL of each dilution was spread on sheep blood agar (OXOID), MacConkey agar (OXOID), Baird Parker medium with egg yolk tellurite emulsion (OXOID), kanamycin aesculin azide agar (OXOID) and incubated at 37 °C. *Pseudomonas* agar base containing CN selective supplement (OXOID) plates were incubated at 25 °C. Growth of bacterial colonies on plates was monitored and recorded after 24 and 48 h of incubation. Bacterial isolates were then identified using standard microbiological procedures, considering production of haemolysin, culture and colonial characteristics, Gram staining, oxidase- and catalase- reaction, coagulase testing and other conventional biochemical tests when needed.

### 2.11. Statistical Analysis

Statistical Analysis Systems version 9.3 (SAS Institute Inc., 1996, Cary, NC, USA) was used for the performance of the statistical analysis. The Shapiro-Wilk Test (PROC UNIVARIATE) was applied to test the normality of the data. The parameters Head, Midpiece, Tail abnormalities and Cytoplasmic droplets did not follow a normal distribution and were normalized by square root transformation. For reasons of clarity, the means and SEM of the not transformed data are presented. To conduct the statistical analysis a repeated measures mixed model (PROC MIXED) was applied. The model included group, time and their interaction as fixed effects and boar as a random effect. Semen sample was defined as the subject of the repeated observations. Covariance structure was chosen based on the values of the Akaike information criterion (AIC). Six models were run with different structures (variance components, compound symmetry, unstructured, first-order autoregressive, first-order ante dependence and Toeplitz) and the model with the least AIC was chosen. Pairwise comparisons where performed with the PDIFF command incorporating the Tukey adjustment. Regarding microbiological data-paired differences between the control and Fe group were calculated for every variable and time point by extracting the values of group Fe from control. The normality of the differences was tested using the Shapiro–Wilk test and normality was evident in all cases. A paired t-test was applied to examine the null hypothesis that the true mean was zero. Statistically significant difference was defined as *p* < 0.05.

## 3. Results

### 3.1. Nanoparticles’ Characteristics

**Figure 2:**
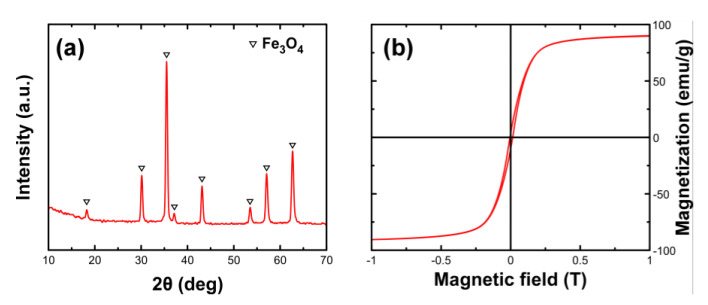
X-ray diffraction (XRD) diagram of the synthesized Fe_3_O_4_ nanoparticles (**a**) and corresponding magnetic hysteresis loop at room temperature (**b**).

The obtained nanoparticles following the described methodology were identified to be iron oxides with inverse spinel structure according to the XRD diagram (Figure 2). However, the saturation magnetization value which approaches 90 emu/g, stands very close to the expected value for magnetite, indicating Fe_3_O_4_ as the dominant phase (Figure 2b). In addition, chemical analysis and specifically the determination of Fe^2+^/Fe^3+^ ratio provides further evidence of the Fe_3_O_4_ presence, which was roughly 43% with the ideal case of Fe_3_O_4_ stoichiometry being 50%. SEM imaging was used to find the geometrical characteristics of the sample (Figure 2a). Nanoparticles appear to show a narrow size distribution with the average diameter of the observed spheres estimated around 42 nm.

### 3.2. Efficiency of the Experimental Process

**Figure 3:**
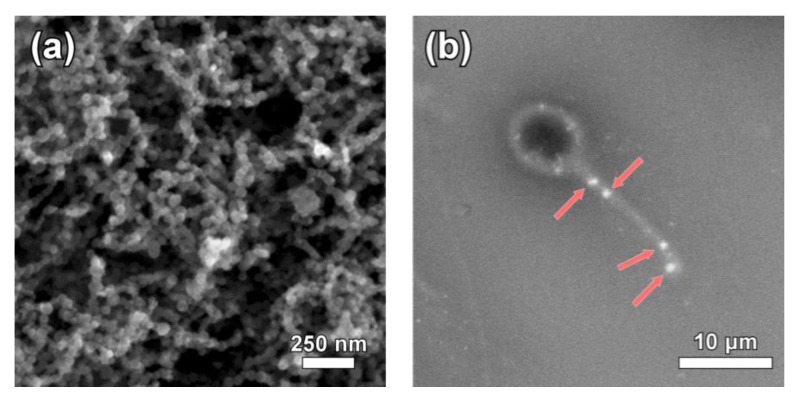
Scanning electron microscope (SEM) image of the synthesized Fe_3_O_4_ nanoparticles (**a**) and representative picture of nanoparticles attachment on a spermatozoon after incubation (**b**).

The efficiency of incubation procedure is illustrated by the attachment of magnetic nanoparticles onto the semen. Figure 3b indicates a representative case were nanoparticles aggregates were located in the tail of an isolated spermatozoon.

### 3.3. Semen Variables’ Assessment

**Figure 4:**
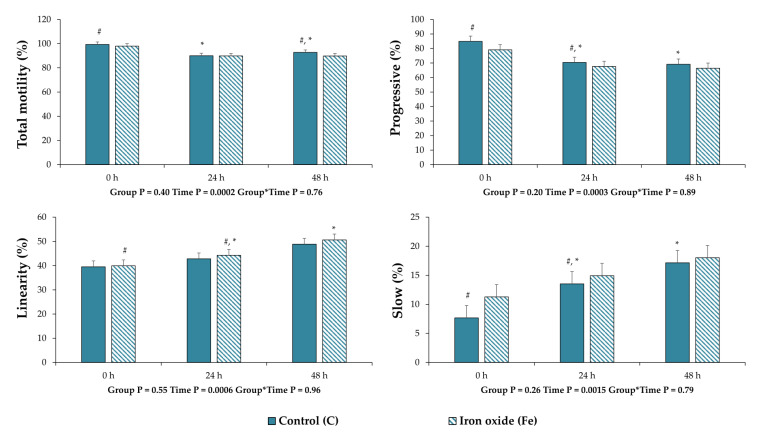
Sperm motility and kinematic parameters of extended boar semen samples at 0, 24 and 48 h of storage post treatment with Fe_3_O_4_ nanoparticles (NPs). Control group (C): extended boar semen samples without any treatment; Iron oxide group (Fe): extended semen with Fe_3_O_4_ NPs (0.192 mg Fe_3_O_4_/mL semen). All the values are expressed as mean ± standard error of the mean (SEM). Different symbols (#, *) denote significant differences between evaluation times within each experimental group.

**Figure 5:**
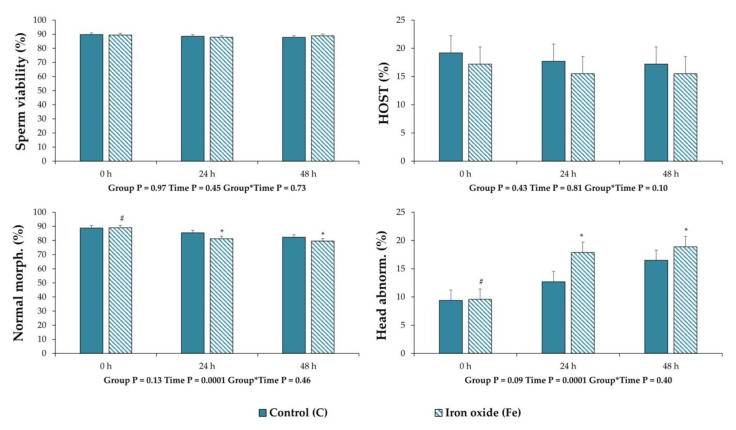
Sperm quality and function variables of extended boar semen samples at 0, 24 and 48 h of storage post treatment with Fe_3_O_4_ nanoparticles (NPs). Control group (C): extended boar semen samples without any treatment; Iron oxide group (Fe): extended semen with Fe_3_O_4_ NPs (0.192 mg Fe_3_O_4_/mL semen). All the values are expressed as mean ± standard error of the mean (SEM). Different symbols (#, *) denote significant differences between evaluation times within each experimental group.

No differences (*p* > 0.05) were observed between the experimental groups in the mean values for all the variables that were assessed in this experiment (Figure 4 and Figure 5, Table 1). However, regarding CASA motility and kinetic parameters, the percentage of total motility (*p* = 0.03) and progressive movement spermatozoa (*p* = 0.03) were less after 24 and 48 h of storage post treatment (0 h) in group C, respectively (Figure 4). For the same group, the percentage of slow movement spermatozoa increased (*p* = 0.03) after 48 h of storage compared to 0 h (Figure 4). In the Fe group, only the LIN decreased (*p* = 0.03) after 48 h of storage compared to 0 h (Figure 4). For the remaining sperm quality and function variables (Table 1 and Figure 5), there were statistical differences only for sperm morphology. Specifically, in the Fe group, the values of spermatozoa with normal morphology decreased (*p* = 0.0001) along the storage period (Figure 5). Regarding the statistical analysis for each category of morphological abnormalities, it was revealed that the deterioration of sperm morphology corresponds only to an increase (*p* = 0.0001) of spermatozoa with head abnormalities in terms of acrosome reacted membrane (Figure 5). Finally, regarding all samples the percentage of DNA fragmentation was 0–1% and no differences were observed between treatments.

### 3.4. Microbiological Results

**Figure 6:**
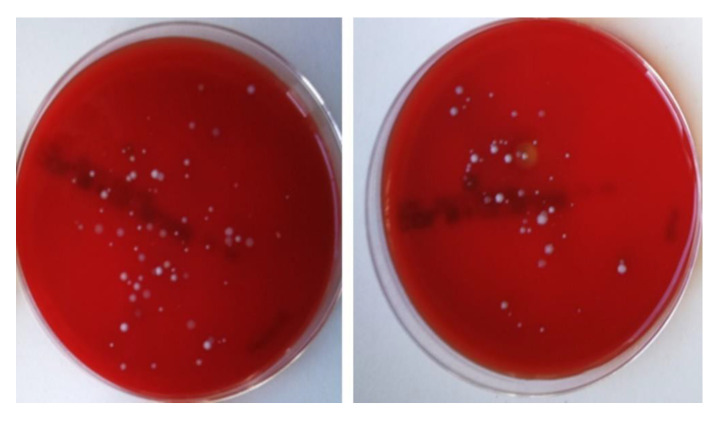
Culture on sheep blood agar [a: control group (C), b: iron oxide group (Fe)]. Reduction of the microbial load after 24 h of incubation (37 °C).

Bacterial growth was present in all semen samples. However, the microbial load varied. The most prevalent bacteria belonged to the Enterobacteriaceae family, *Staphylococcus* spp., *Enterococcus* spp. and *Pseudomonas* spp. The latter was found not to be affected by the examined NPs when detected at the specific concentration (data not shown). Total bacterial count in boar semen was respectively low and the number of cfu/mL demonstrated a wide range of microbial load among samples (from 45 to 1855, min–max, respectively). Treatment with Fe_3_O_4_ NPs did not eliminate bacterial content (Figure 6). However, a statistically significant reduction of the microbial load of semen was evident (*p* = 0.03) (Table 2). Among the other detected bacteria staphylococci tended to be less on Fe group compared to control, while Enterobacteriaceae (*Enterobacter* spp., *E. coli*, *Proteus* spp) and *Enterococcus* spp. had no significant difference from the control group (Table 2).

## 4. Discussion

Despite the potential biological benefits of NPs, nanotoxicity and its impact on cells’ health is a concern [14]. Previous studies reported that Fe_3_O_4_ NPs affect rainbow trout sperm [24], whilst titanium dioxide TiO_2_ NPs negatively affect mouse gene expression of Leydig cells, as well as semen quality parameters [25]. No studies were found to report the effects of Fe_3_O_4_ NPs on boar semen. Thus, the first aim of this study was to perform a full laboratory assessment of boar semen processing with iron oxide NPs. This study was based on previous findings of our research team [15], that explored the minimum inhibitory concentration of Fe_3_O_4_ NPs (0.192 mg/mL semen) and the appropriate co-incubation time of semen with NPs (30 min). However, the present study assessed the full profile regarding boar sperm characteristics.

The process of fertilization is a complex of sequencing events, involving the normal movement of the spermatozoa to reach the oviduct, the approach to oocyte, the sperm acrosome reaction, the sperm penetration into the ooplasm, the merging of the gametes, the fusion of the pronuclei and the intermingling of the paternal and maternal chromosomes. Low semen quality, as expressed by variables from semen analysis, is a common cause of subfertility or infertility [24]. This study provided a protocol for co-incubation of sperm with NPs, regarding both time and concentration, which had no detrimental effect on semen parameters. No effects were observed regarding sperm viability, morphology, membrane functionality, DNA integrity and CASA analyzed kinematics. This is an important finding, as it realizes the use of Fe_3_O_4_ NPs in boar semen handling with no toxicity. The sperm parameters analyzed in this study are of paramount importance for the fertilizing capacity of boar semen. Many researchers highlighted that motility is better correlated to field fertility compared to other kinetic parameters [26,27]. Moreover, Broekhuijse et al. [28,29] showed that CASA parameters, like progressive motility, BCF and VCL, could be related to farrowing rate, while the total number of born piglets could be affected by total motility, ALH, VSL and VAP [26,27]. In accordance with these findings, Holt et al. [30] showed that the VSL could positively affect the litter size. In vitro fertility of boar sperm has been positively correlated with progressive motility, VAP and VSL, and negatively correlated with STR, LIN and ALH [31]. Also, it is well accepted that the more diagnostic tests performed (such as the assessment of sperm morphology, motility and chromatin integrity), the better the prediction of in vitro fertility that can be achieved [32,33]. None of the above-mentioned sperm parameters were affected in our study. It seems that the restricted period of sperm co-incubation with Fe_3_O_4_ NPs and the gentle removal of them with a magnetic field, protected boar spermatozoa from NPs toxicity. This is in agreement with previous reports, that suggest a hypothesis that the time of interaction between semen and NPs can be crucial regarding their potential toxic activity [34,35]. In accordance with this scientific hypothesis, a significant decrease of VCL, VSL and VAP was observed after a prolonged (24 h) incubation of rainbow trout semen with Fe_3_O_4_ NPs [24].

In the Fe group, the value of spermatozoa with acrosome-reacted membrane was increased during storage time, which was not the case for the control group. However, even if this effect was statistically significant, the reported numerical values are not indicative of an important biological consequence. This finding could be attributed to the presence of the examined NPs. It is known that NPs penetrate the cells’ membranes affecting their physiology [36]. Although it was not within the purposes of the present study, according to the literature, the most prevalent mechanism of action of nanoparticles is related to the induction of oxidative stress [37]. Subsequently, the increased production of ROS can irreversibly affect the membranes of spermatozoa and can be involved in sperm capacitation [38], leading to the perturbation of the acrosome membrane’s integrity [39]. Therefore, the reported increase in spermatozoa with reacted acrosome membrane could be an undesirable characteristic for extended liquid semen used in AI programs, but it could be a perspective for IVF protocols, in which the induction of acrosome reaction is a prerequisite.

Semen extenders are cell culture media and thus an ideal environment for bacteria proliferation. There are directives from the European Union [40] and from national governments that specify the antibiotics’ category and dose as semen extenders’ additives. A variety of antibiotic compounds have been used to control microbial contamination in extenders. In farm animals’ AI, including boars, streptomycin and penicillin are the most widely used antibiotics [41]. Additionally, antibiotics, like gentamicin, linco-spectin and clindamycin have been successfully implemented in different semen extenders [42,43]. During the last decades, the bacterial resistance to antibiotics has been a serious problem to humans’ as well as animals’ health. Moreover, some antibiotics, at certain concentrations, may have a direct detrimental effect on spermatozoa [44]. Contemporary studies have proved that some antimicrobials may have a deleterious effect on bull [45] and equine [46] spermatozoa. In this aspect, NPs have been an interesting alternative for the scientists. In the present study, the examined iron oxide NPs had no toxic effect on boar spermatozoa and showed a slight antibacterial effect, although not for all bacteria species. Boar semen contaminants were present in all samples of the study, but the microbial load varied between them. Total bacterial count for aerobic mesophiles was up to 1.8 × 10^3^, while Gączarzewicz et al. [4] reported findings up to 360 × 10^6^. Despite the different degrees of contamination, a significant reduction regarding the total microbial count was observed in the presence of Fe_3_O_4_ NPs compared to control after 24 and 48 h of incubation. Given the initial low microbial load, this reduction suggests a promising result for the use of Fe NPs in heavier bacterial contaminations. After culture on selective media, the antimicrobial activity of Fe_3_O_4_ NPs demonstrated variation depending on the strain. The predominant bacteria on the samples were *Staphylococci* and Enterobacteriaceae, similar to studies previously reported [4,47]. Treatment with Fe_3_O_4_ NPs reduced the number of viable *Staphylococci*, though had a minimum effect on the Gram negative Enterobacteriaceae. In this field, reports demonstrate the bactericidal properties of iron oxide NPs (50 and 100 μg/mL) against *Shigella dysentery* and *Escherichia coli* [48] as well as their antimicrobial effects on antibiotic-resistant strains of *E. coli* [49]. Others report that iron NPs have only moderate antimicrobial action against *Escherichia coli* and *Bacillus subtilis* but remain potentially useful for application in the pharmaceutical and biomedical industry [50]. Studies have reported NPs to have excellent antimicrobial resistance properties and the ability to inhibit the formation of bacterial biofilms [51,52], which favor their use in drug-delivery systems. Concerning their mechanism of action, under specific conditions iron oxide nanoparticles may provide significant antimicrobial activity in contact with common bacteria. The interaction is stronger when nanoparticles’ surface is positively charged and when it involves the presence of both Fe^2+^ and Fe^3+^. Both properties are met in uncoated Fe_3_O_4_ nanoparticles distributed in the pH of biological environment. It has been reported that positively-charged Fe_3_O_4_ nanoparticles indicate stronger interaction with bacteria while the addition of surfactants counterbalances surface charge and limits such tendency [53]. An occurring mechanism involves the attachment of Fe_3_O_4_ nanoparticles on the bacteria membrane, and the dissolution of Fe^2+^ and Fe^3+^ at their interface which initiates the generation of reactive oxygen species. This triggers hydrogen peroxide release and production of free radicals through a Fenton reaction:

Fe^3+^ + H_2_O_2_ → Fe^2+^ + OH^−^ + OH^•^

Fe^2+^ + H_2_O_2_ → Fe^3+^ + HO_2_^•^ + H^+^

The presence of toxic free radicals on bacteria membrane causes its electrostatic modification inducing chemical stress that causes the damage of the bacteria unit. The antimicrobial potential of Fe_3_O_4_ nanoparticles is preserved until their surface gets fully oxidized to *γ*-Fe_2_O_3._ Armijo et al. [54] findings suggest that Fe_3_O_4_ NPs are potential alternatives to silver NPs in several antibacterial applications minimizing the cost and enhancing microbial inactivation and elimination. The results of our research are aligned with these findings, reinforcing the future utilization of the antibacterial activity of Fe_3_O_4_ NPs as a new perspective to prevent bacteria in semen.

In conclusion, the Fe_3_O_4_ NPs examined are a potential useful and effective semen supplementation with antibacterial properties. Moreover, the combination of NPs with conventional antibiotics could enhance their antibacterial action and thus reduce the dose demanded. It is suggested that further studies regarding the oxidative status of boar semen treated with Fe_3_O_4_ NPs should be carried out to investigate the mechanism of action on boar spermatozoa.

## Figures and Tables

**Table 1:** Computer-assisted sperm analyzer (CASA) kinematic parameters (mean ± SEM) of extended boar semen samples at 0, 24 h and 48 h of storage post treatment with Fe_3_O_4_ NPs.

Variable	Group C			Group Fe			*p* Value		
	0 h	24 h	48 h	0 h	24 h	48 h	Group	Time	G*T
Rapid (%)	68.83 ± 4.68	57.95 ± 4.68	52.17 ± 4.68	64.57 ± 4.68	51.26 ± 4.68	50.19 ± 4.68	0.26	0.0042	0.88
Medium (%)	22.85 ± 2.01	18.60 ± 2.01	23.44 ± 2.01	22.36 ± 2.01	18.72 ± 2.01	21.60 ± 2.01	0.65	0.09	0.88
VCL (μm/sec)	64.65 ± 3.14	62.66 ± 3.14	55.62 ± 3.14	61.57 ± 3.14	58.14 ± 3.14	55.51 ± 3.14	0.32	0.06	0.77
VSL (μm/sec)	25.02 ± 2.52	28.39 ± 2.52	30.39 ± 2.52	24.04 ± 2.52	27.07 ± 2.52	29.66 ± 2.52	0.63	0.10	0.99
VAP (μm/sec)	47.48 ± 3.09	43.32 ± 3.09	36.72 ± 3.09	46.42 ± 3.09	41.82 ± 3.09	37.78 ± 3.09	0.84	0.0107	0.91
ALH (μm)	2.75 ± 0.16	2.52 ± 0.16	2.29 ± 0.16	2.56 ± 0.16	2.30 ± 0.16	2.28 ± 0.16	0.29	0.07	0.80
BCF (Hz)	5.49 ± 2.45	5.57 ± 2.45	5.32 ± 2.45	5.26 ± 2.45	5.52 ± 2.45	5.14 ± 2.45	0.43	0.45	0.93
STR (%)	49.07 ± 2.85	49.76 ± 2.85	53.84 ± 2.85	52.81 ± 2.85	51.12 ± 2.85	55.69 ± 2.85	0.32	0.26	0.91
WOB (%)	72.90 ± 1.77	75.10 ± 1.77	75.77 ± 1.77	74.39 ± 1.77	76.87 ± 1.77	76.27 ± 1.77	0.39	0.31	0.93
Hyper (%)	0.74 ± 0.43	1.08 ± 0.43	0.84 ± 0.43	0.93 ± 0.43	0.99 ± 0.43	1.31 ± 0.43	0.39	0.16	0.53

Group C: untreated extended semen sample, and Group Fe: extended semen sample treated with Fe nanoparticles. Time points 0, 24 and 48 h: storage time post removal of Fe_3_O_4_ NPs. Rapid Medium: rapid, medium movement spermatozoa (%; 25<medium<45<rapid μm/sec); VCL: curvilinear velocity (μm/sec); VSL: straight line velocity (μm/sec); VAP: average path velocity (μm/sec); STR: straightness (VSL/VAP × 100); WOB: wobble (VAP/VCL × 100); ALH: amplitude of lateral head displacement (μm); BCF: beat/cross-frequency (Hz); Hyper.: hyperactive spermatozoa (%). G*T: Group*Time interaction.

**Table 2:** Microorganisms (cfu/mL) isolated from boar semen samples 24 h and 48 h after incubation on blood agar and selective culture media.

Variable	Control	Fe	Difference (Control-Fe)	*p*-Value
Blood Agar 24 h	558 ± 455	443 ± 381	115 ± 103	0.03
Blood Agar 48 h	779 ± 651	616 ± 515	164 ± 150	0.03
*Staphylococcus* spp	314 ± 411	251 ± 380	63 ± 92	0.06
*Enterococcus* spp	5.4 ± 7.6	4.5 ± 8.7	0.9 ± 7.9	0.72
Enterobacteriaceae	69.9 ± 106.6	60.1 ± 86.5	9.8 ± 27.5	0.29
